# Shaping of the alveolar landscape by respiratory infections and long-term consequences for lung immunity

**DOI:** 10.3389/fimmu.2023.1149015

**Published:** 2023-04-04

**Authors:** Lucia Rodriguez-Rodriguez, Laurent Gillet, Bénédicte Machiels

**Affiliations:** Laboratory of Immunology and Vaccinology, Faculty of Veterinary Medicine, Fundamental and Applied Research for Animals & Health Research Unit (FARAH), ULiege, Liege, Belgium

**Keywords:** respiratory viruses, lung immunity, alveolar macrophages, niche imprinting, trained immunity, AM ontogeny

## Abstract

Respiratory infections and especially viral infections, along with other extrinsic environmental factors, have been shown to profoundly affect macrophage populations in the lung. In particular, alveolar macrophages (AMs) are important sentinels during respiratory infections and their disappearance opens a niche for recruited monocytes (MOs) to differentiate into resident macrophages. Although this topic is still the focus of intense debate, the phenotype and function of AMs that recolonize the niche after an inflammatory insult, such as an infection, appear to be dictated in part by their origin, but also by local and/or systemic changes that may be imprinted at the epigenetic level. Phenotypic alterations following respiratory infections have the potential to shape lung immunity for the long-term, leading to beneficial responses such as protection against allergic airway inflammation or against other infections, but also to detrimental responses when associated with the development of immunopathologies. This review reports the persistence of virus-induced functional alterations in lung macrophages, and discusses the importance of this imprinting in explaining inter-individual and lifetime immune variation.

## Introduction

Macrophages are highly specialized phagocytes that play a central role in both tissue homeostasis and inflammation. Indeed, they play a supporting role in tissue development and repair or during the innate response developed against pathogens. However, they also contribute to the pathophysiology of multiple diseases including cancer and various inflammatory disorders. Tissue-resident macrophages are thus extremely heterogeneous in origin and function and possess a unique transcriptome that allows them to fulfil niche-specific functions. Understanding the ontogeny and the different processes that regulate the fate of tissue-resident macrophages is fundamental to enable the design of future intervention strategies to modulate their functions at specific sites. In this review, we focus on alveolar macrophages (AMs) and discuss recent advances in the field of AM ontogeny, heterogeneity, and function in the context of health and disease. Considering respiratory viral infections as critical environmental factors, we highlight the complexity of the AM population over the lifespan and during successive episodes of infection, resulting in profound changes in the response of these cells to subsequent lung injury.

## Alveolar macrophage identification, location and function

AMs reside in the airways and the alveoli and represent the most abundant population of pulmonary macrophages (1). AMs perform key functions such as surfactant and pathogen clearance, and also orchestrate the response to inflammation and tissue repair (2, 3). These innate cells are professional phagocytes and indispensable housekeepers of homeostasis. Indeed, a reduction in the number of AMs and/or an alteration in their functionality leads to alveolar proteinosis, a disease that results in impaired oxygen exchange due to surfactant accumulation (4). Furthermore, AMs contribute to lung regeneration by increasing their local proliferation and expression of angiogenesis-related genes (5).

AMs are characterized by their high autofluorescence and can be identified in mice as CD11b^-^CD64^+^CD169^++^CD11c^++^SiglecF^++^ (6, 7). However, it is important to note that the expression of some of these markers is shared by other immune cells in the lung. For example, CD64, besides being expressed by AMs and interstitial macrophages (IMs), is also expressed by inflammatory conventional type 2 dendritic cells (cDC2s) that infiltrate the lung during inflammation (8). In addition, specific subtypes of IMs are reported to express the CD169 marker (9–11). Although SiglecF is downregulated on MO-derived AMs (MO-AMs) (12), co-expression of CD11c and SiglecF is the most reliable marker combination for the identification of AMs in mice (7). In contrast, AMs in humans lack expression of Siglec8, the functional paralog of SiglecF and can be identified as CD11b^+^HLA-DR^++^CD206^+^CD169^+^ cells (13).

AM location within the lumen of the alveoli makes these cells one of the first immune cells to come into contact with inhaled particles and pathogens. The exposed nature of the alveolar niche requires AMs to maintain a key balance between tolerogenic and inflammatory responses. This balance should avoid tissue damage, while facilitating a fast and efficient response against pathogens. Studies in the 80s-90s provided the first evidence of a role for AMs in controlling pulmonary immune responses (14), such as the regulation of T cell responses (15–18) and modulation of DCs (19, 20). Both human and rodent AMs affect T cell proliferation *in vitro* (15, 16). Furthermore, depletion of AMs *in vivo* increases the proliferative capacity of lung T cells (14–17). In particular, the release of soluble mediators by AMs, such as nitric oxide (NO), renders T cells unable to proliferate in response to interleukin (IL-)2 stimulation (16, 21). Moreover, AMs may also modulate DC function by reducing DC migration (19), but also by secreting transforming growth factor-beta (TGF-β), prostaglandin E2 (PGE2) and NO, which can alter DC function and maturation (20–24).

In addition to being guardians of lung homeostasis by tempering the activation of other immune cells, AMs contribute directly to protection against pathogens. As such, AMs play a critical role in the early responses to infections: they are key sentinels of fungal infections (25) and they are the main Interferon-alpha (IFNα)-producing cells after pulmonary infection with RNA viruses (26). Furthermore, AMs are initiators of the activation and recruitment of natural killer (NK) cells during RSV infection (27), and contribute to host survival by maintaining lung function during influenza infection (28). Beside viruses, AMs also mediate bacterial clearance (29, 30) and have an anti-inflammatory protective role against several bacterial infections, including pneumococcal pneumonia (31). Indeed, the contraction of the AM population during the acute phase of viral infections may also have a significant impact on susceptibility to secondary bacterial infections (32). In addition to their role during homeostasis and in early responses to infection, AMs also contribute to the resolution of inflammation. AMs do this by efferocytosis of apoptotic cells, in particular neutrophils (33), which can lead to the release of anti-inflammatory mediators such as TGF-β, PGE2 and platelet activating factor (PAF) (34). Furthermore, ingestion of apoptotic cells may upregulate the expression of death receptor Fas ligand (FasL) on AMs (35). FasL on AMs can induce apoptosis of bystander leukocytes, but also self-induced cell death in AMs (36), a process that appears to contribute to bacterial clearance and resolution of lung infection (35, 37). Overall, AMs act as key regulators of homeostasis, but also of the initiation and resolution of immune responses in the lung.

The non-inflamed lung is estimated to contain a single AM for every three alveoli (38). In order to compensate for this numerical disadvantage, AMs have been observed to crawl into and between alveoli using the pores of Kohn, a process that seems to be key in the control of bacterial infections (39). Indeed, if AM chemotaxis is impaired, AMs are unable to clear inhaled bacteria, resulting in increased neutrophil infiltration and release of pro-inflammatory cytokines (39). It has been proposed that this numerical disadvantage of AMs may act as a threshold for the recruitment of neutrophils when bacterial loads exceed the clearance capacity of AMs (35, 39). In parallel to the potential motility of AMs between alveoli, AMs have been speculated to infiltrate the lung draining lymph nodes (dLN) where they deliver phagocytosed particles (40), but also pathogens to B cell regions (41). However, this claim appears to be controversial as many other studies have contrarily concluded that AMs do not migrate to dLN (19, 26).

In addition to the highly motile population of AMs, another study has also identified a population of AMs attached to the epithelium that is immobile and cannot be recovered from the bronchoalveolar lavage (BAL) (38). This population of AMs communicate with neighbouring AMs in other alveoli through synchronized Ca2+ waves across the epithelium. This interalveolar communication seems to be important to coordinate the release of immunosuppressive signals during LPS-induced inflammation (38).

## Neighbouring macrophage populations in the lung: How do they differ from alveolar macrophages?

In addition to AMs, IMs constitute another population of lung macrophages that are present in lower numbers. This population can be identified in mice as CD11b^+^Ly6G^-^MHCII^+^CD64^+^CD24^-^ (7) and in humans, as CD11b^+^HLADR^++^CD206^+^CD169^-^ (13) (**Table 1**).

**Table 1:** Identification of alveolar macrophages and interstitial macrophages in mouse and human according to specific surface markers expression, origin, location and function.

	AMs		IMs	
	Mouse	Human	Mouse	Human
**Markers**	CD64	CD206	CD64	CD206
	CD11c	CD11b	CD11b	CD11b
	HLA-DR**	HLA-DR	HLA-DR	HLA-DR
	CD169	CD169	CD169*	
	SiglecF		Lyve1*	
			CD206*	
**Origin**	**FL-MOs** CD45+C-kit−F4/80loCD11bint Ly6G−Ly6ChiMHCII−CD11c−	**Fetal precursors** CD34-Lin-CD116+CD64-CD115+	YS-MACs BM-MOs	
	BM-MOs***	BM-MOs***		
**Location**	Alveolar		Bronchial interstitium close to blood vessel	
	Interalveolar		Alveolar Interstitium close to nerves	
**Function**	Surfactant regulation		Support for structural compartment	
	Clearance of pathogen, particles, apoptotic cells		Regulation of inflammation (source of IL-10 production)	
	Early response to infection		Control of fibrosis	
	Regulation of T cell and DC responses		Regulation of T cell responses	
	Modulation of allergic responses during allergen sensitization and challenge		Modulation of allergic responses during allergen sensitization	

*Different levels of expression of these markers delineates specific macrophages subtypes.

**This marker can be upregulated during inflammation or during differentiation of MOs into AMs.

***Present during life and inflammatory conditions.

IMs localize in the lung interstitium and play important roles in antigen presentation, tissue remodelling, but also in the modulation of DC function to prevent house dust mite (HDM)-induced airway allergy (42). While IMs modulate DC function during both the sensitisation and challenge phases of allergic airway inflammation (43), it is important to note that MO-derived DCs also play a role by affecting the challenge phase of HDM-induced airway allergy (44). Besides, AMs modified in the airways after a gammaherpesvirus infection also appear to be necessary and sufficient to protect against HDM-induced allergic airway inflammation (45).

IMs are phenotypically and functionally different from AMs. For example, AMs have increased phagocytic (46, 47), microbicidal and tumor cytotoxic activity (48) compared to IMs. However, IMs show increased expression of MHCII (42, 46) and seem to be more effective in the induction and maintenance of specific immune responses (46, 48). Furthermore, AMs and IMs show different qualitative responses to TLR4 stimulation *in vivo*, with AMs exhibiting a delayed response compared to IMs (49). They also display different quantitative responses *in vitro*, with AMs producing increased levels of tumor necrosis factor alpha (TNF-α), and IMs of IL-6 and IL-10 (50).

Transcriptionally different populations of IMs have been identified in specific sub-tissular niches in the lung but also in other tissues. An initial characterization of IM diversity using RNAseq identified three different subpopulations: CD11c^-^CD169^hi^CD206^hi^Lyve-1^hi^MHCII^lo^ (IM1), CD11c^-^CD169^hi^CD206^hi^Lyve-1^lo^MHCII^hi^ (IM2), and CD11c^lo^CD169^lo^CD206^lo^MHCII^hi^ (IM3) (9). These populations had a slow turnover, and the population IM3 showed an increased replenishment from circulating precursors compared to population IM2 and especially IM1. Later on, Chakarov et al. further characterized IM diversity using single-cell mRNA and identified only two main subpopulations of IMs: Lyve1^hi^MHCII^lo^ and Lyve1^lo^MHCII^hi^, that reside specifically adjacent to blood vessels and to nerve bundles and endings, respectively (11). These populations were described as separate lineages that originate from recruited MOs and have different functionalities. On the one hand, Lyve1^lo^MHCII^hi^ macrophages have increased capacity to activate and to induce proliferation of CD4^+^ T cells, but also to promote T regulatory cell differentiation. On the other hand, Lyve1^hi^MHCII^lo^ macrophages produce increased levels of IL-10 and are critical for controlling the development of fibrosis by reducing collagen deposition, immune cell infiltration and tissue inflammation (11). The described populations correspond also to CD206^+^ (Lyve1^hi^) and CD206^-^ (Lyve1^lo^) IM populations depicted in another concomitant study (51). While CD206^+^ IMs are located in the bronchial interstitium close to blood vessels, CD206^-^ IMs are located in the alveolar interstitium (51). Furthermore, similar to what Gibbings et al. (9) had previously observed for IM1 population, CD206^+^ IMs had also increased self-maintenance potential. In addition, while CD206^+^ IMs produced increased levels of immunoregulatory cytokines, CD206^-^ IM had a typical antigen-presenting cell profile (51). Afterwards, another study also identified a population of CD169^+^Lyve^lo^MHCII^hi^ lung resident macrophages that localized around the airways in close proximity to sympathetic nerves and played an important role in regulation of inflammation (10). This population of IMs corresponds to that described by Chakarov et al. and has a similar transcriptomic signature to the macrophage population identified by the scRNA-sequencing of the whole lung performed by Cohen et al. (52). However, while Chakarov et al. established that this population of IMs originate from circulating MOs, Ural et al. propose that they are yolk sac (YS)-derived cells that are not replaced by circulating MOs at steady state (10).

## Origin of alveolar macrophages

**Figure 1:**
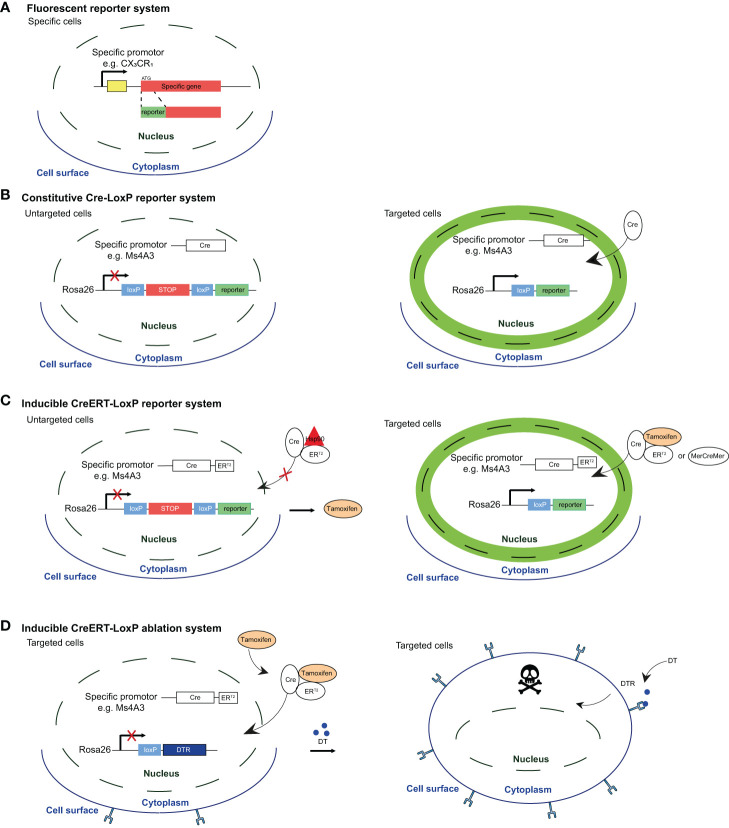
Fluorescent reporter system and Cre-based mouse models for reporter labeling and cell ablation. **(A)** Reporter mice were developed by inserting a recombinant gene encoding a fluorescent protein (e.g. GFP, RFP) under the expression of a specific promoter. A general drawback of these reporter system is that fluorescence intensity is directly associated with the activity of the promoter. Therefore, one limitation is that expression level may fluctuate over time in lineage-tracing or during inflammation. Moreover, overlapping expression of different markers by EM-AMs and MO-AMS makes the distinction between these subsets quite challenging. **(B)** In the second generation of reporter mice, a Cre recombinase-encoding gene is inserted under the control of a specific promoter, while a fluorescent protein-encoding gene -preceded by a STOP codon flanked by loxP sites (flaxed) is inserted under the control of constitutively active promoter (e.g Rosa26). This system allows to create reporters with fluorescence of choice (e.g. GFP, RFP, mcherry, TdTomato,...), and labels cells in a on/off manner instead of depending on the fluctuating promoter activity. However, these fate-mapping models do not allow to discriminate the homeostatic contribution of MOs to the AM pool during life from the one specifically recruited in disease. **(C)** Inducible reporter mice, in wich the Cre-loxP system is activated by exogenous inducer (tamoxifen) that allows to assess the specific contribution of MOS to the AM pool at a given time. Indeed, upon exogenous administration, tamoxifen binds to estrogen-receptor (ER^T2^ or MercreMer) leading to the dissociation of the complex that sequesters Cre recombinase in the cytoplasm and to the subsequent translocation into the nucleus, allowing reporter expression Interestingly, after tamoxifen treatment has been interrupted, short-lived cells rapidly loose the signal while long-lived cells remain labeled over time. **(D)** Cre-LoxP systems allowing for targeted cell ablation by combining cell-specific CreER^T2^ expression with the induction of a cellular killing mechanism However, the results could be skewed by off-target effects or conversely, good Cre-driving promoter candidates may show only low activity, and present as poor Cre inducers with inadequate recombination efficiency. Figure adapted from Karen De Vlaminck PhD thesis (Bioengineering Sciences VUB).

**Figure 2:**
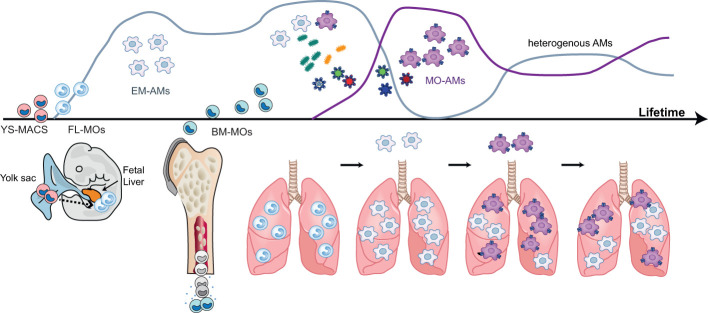
Ontogeny and development of alveolar macrophages during life. During the pre- and post-natal period, alveolar macrophages (AMS) have an embryonic ontogeny as they derive from successive waves of precursors originating first from the yolk sac (YS-MACS) and then from the fetal liver (FL-MOs). These AMs of embryonic origin display a high proliferative capacity which allows them to occupy the expanding alveolar niche compartment and to self-sustain with little or no contribution from bone marrow-derived monocytes (BM-MOs) However, during life and the ageing process, BM-MOs will gradually infiltrate the lung, differentiate into AMs and progressively contribute to the AM population. The contribution of BM-MO to the pool of AMS increases following episodes of inflammation and/or respiratory infections inducing the depletion of the alveolar niche and the recruitment of MOs into the alveoli. The magnitude, persistence and functional consequences of this BM-MO contribution to the AM pool are context-dependent and the mechanisms dictating this heterogeneity are not fully elucidated. Figure adapted from Karen De Vlaminck PhD thesis (Bioengineering Sciences VUB).

In mice, the use of fate mapping models including *Runx1*MercreMer (53–55), *Csf1r*MercreMer (53–56), *Cx3cr1*creER^T2^ (57), *Kit*MercreMer (58), *S100a4cre*-RosaTdT (53, 59), *Flt3cre*-RosaTdT/YFP (56, 59), *Tie2*MerCreMer (56) and Ms4a3CreER^T2^-RosaTdT (60), together with irradiated chimeric mice (61), parabiosis (55, 59, 61) and adoptive transfer models (57, 59, 61), has provided insightful information about the developmental biology of tissue-resident macrophages (**Figure 1**). Based on these models, it has been proposed that AMs, like Kupffer cells or microglia, develop from erythro-myeloid progenitors (EMPs) (53, 56). In mice, early EMPs c-Myb^-^ are generated in the YS at day E7.5 and give rise to YS macrophages (YS-MACs) by day E9 (**Figure 2**). These “primitive” YS-MACs seed embryonic tissues, including the lung, prior to the emergence of fetal liver (FL) haematopoiesis at day E10.5. At this stage, late c-Myb^+^ EMPs migrate and colonize the FL and give rise to myeloid c-Myb^+^Csf1r^+^ progenitors that differentiate into FL-MOs. These FL-MOs differentiate into tissue resident macrophages from E13.5 onwards, with the exception of microglia which are derived directly from YS-MACs (53). Interestingly, the transition of late EMPs into FL-MOs seems to be key to enhance the developmental potential of these precursors to differentiate into tissue-resident macrophages (62).

The origin of macrophages in the lung follows different developmental waves. Primitive YS-MACs seed the lung in a first wave around day E10.5 (53, 61). Two days later, incoming FL-MOs expressing Mac2 populate the lung and enter the alveoli during the first week of post-natal life, giving rise to AMs (63). During the first weeks of birth, incoming bone marrow MOs (BM-MOs) give rise to the definitive population of IMs located throughout the interstitium (55). Interestingly, the primitive population of YS-MACs seems to persist after birth in peripheral and perivascular locations. According to previous studies, FL-MOs end up replacing primitive YS-MACs over time (53, 61). However, YS-MACs do not undergo apoptosis after the emergence of FL-MACs and otherwise appear to be diluted by the new incoming populations, leaving a residue of approximately 2-3% of these cells in the lung after birth (53).

It has been proposed that the differentiation of FL-MOs into AMs follows consecutive waves of differentiation, in which FL-MOs transit into a pre-AMs stage (Ly6C^int^CD11b^hi^F4/80^int^CD64^int^) between E18-PND1 and differentiate into mature AMs 3 days after birth (61). Proliferation and differentiation of AMs depend on both intrinsic and extrinsic signals (**Figure 2**), among which Granulocyte-macrophage colony-stimulating factor (GM-CSF), TGF-β and Peroxisome proliferator-activated receptor gamma (PPARγ) are of crucial importance (59, 61, 63–67). Interestingly, the isolation of FL-MOs and their *in vitro* culture in the presence of GM-CSF, allows the generation of long-lived immature ‘AM-like’ cells, which develop into mature and functional AMs when transplanted *in vivo* (68). In the lung, GM-CSF is expressed by alveolar epithelial type II cells (AEC2), basophils and innate lymphoid cells (52). On the other hand, TGF-β is mainly expressed by epithelial cells, endothelial cells and especially AMs, which allows this cytokine to act on an autocrine manner (63). Both GM-CSF and TGF-β converge in inducing PPARy expression, and they are essential for the maintenance of AMs and their imprinted signature (63, 65). Furthermore, a recent study has described the important role of Arachidonate 15-Lipoxygenase (ALOX15) pathway in neutrophil-dependent AM instruction during lung development (69). According to this study, perinatal neutrophil secretion of 12-Hydroxyeicosatetraenoic acid (12-HETE) *via* ALOX15 pathway, is able to imprint AM self-renewal programming for the long-term, leading in absence of ALOX15, to the presence of senescent AMs with reduced proliferative capacity in adult mice (69).

Humanized mouse models have also shown that AMs develop from EMP-like precursors (70). In particular, it has been proposed that fetal CD34^-^Lin^-^CD116^+^CD64^-^CD115^+^ precursors migrate into the lung, in a possible CX3CR1-dependent manner, and then differentiate into AMs in response to GM-CSF and Macrophage colony stimulating factor (M-CSF) (70). However, it is still unclear whether these EMP-like precursors differentiate directly into AMs, or if they first differentiate into FL-MOs CD116^+^CD64^+^ and then into AMs. MO differentiation into AMs in humans seems to follow a step wise differentiation, in which circulating CD16^-^CD14^+^ MOs give rise to extravasating MHCII^hi^ MOs, then differentiate into immature CD206^+^CD169^-^ AMs, and eventually mature into CD206^+^CD169^+^ AMs (71). A similar transition of MOs into macrophages through MHCII upregulation on MOs has also been described in mice during the renewal of macrophages populations at steady-state in the peritoneum (72), but also in the gut (73). Furthermore, in the lung, an intermediate population of pre-AMs, that shares a similar phenotype to IMs, has also been reported to follow the differentiation of MOs into AMs (74).

## Niche instruction of alveolar macrophages

**Figure 3:**
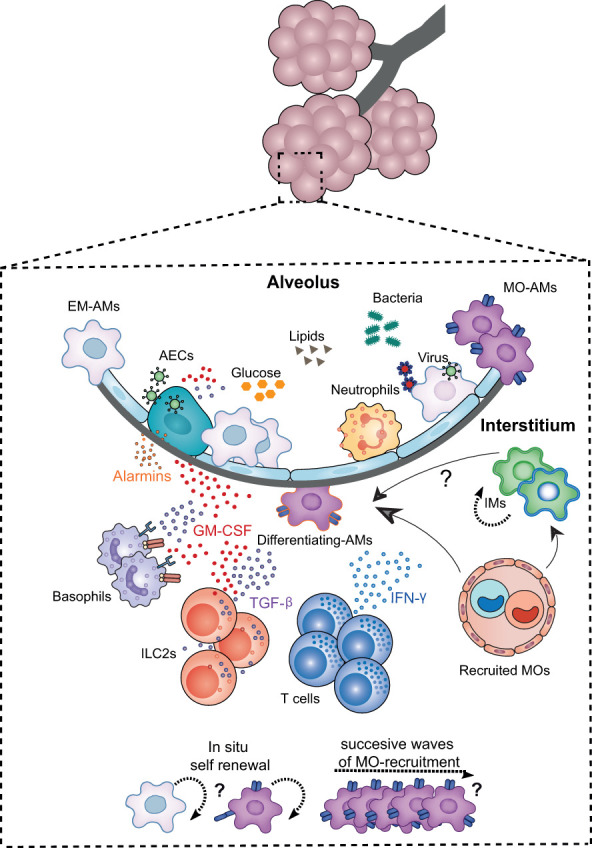
Niche microenvironment is a major determinant governing AM development, proliferation and activation. The developmental dynamics of AMs and their plasticity are intimately linked to signals from the local environment. The alveolar niche, in addition to supplying the structural scaffold necessary for the development of AMs, harbours key cellular actors that provide the trophic factors determining the differentiation, maturation, proliferation or functional polarisation of these cells. Among the niche cells identified *in the steady state*, alveolar epithelial cells (AECs), innate lymphoid cells type 2 (ILC2s), and basophils imprint AM identity and function, both at birth and throughout life, notably via the secretion of GM-CSF. TGF-β. *Upon lung infection*, the EM-AM population is partially depleted making the alveolar niche more accessible to BM-MO infiltration and differentiation into AMs in a similar GM-CSF and TGF-B-depend manner. The contribution of MOS to the AM pool is highly dependent on the type of inflammation, the extent of depletion, the turnover rate of AMs and IMs in the lung, and the presence of other imprinted niche cells such as altered ILC2s and/or tissue-resident T cells. In particular, the combination of chemokines and cytokines such as IFN-γ released during inflammation, and the altered availability of trophic factors and nutrients may determine the differentiation of MOs into AMs, their polarisation as well as their renewal capacity.

The alveolar niche represents the physical and nurturing scaffold (75) where AMs reside. The niche not only provides instructions for AM differentiation during pre- and post-natal development, but also during adult life, ensuring the maintenance of their hyporesponsive state and function. The niche cells identified so far that are involved in this process are: epithelial cells, innate lymphoid cells group 2 (ILC2s) and basophils, which produce different factors that regulate and imprint AM identity (**Figure 3**).

## Epithelial cells

Alveolar epithelial cells (AECs) are key components of the alveolar niche. In particular, production of GM-CSF by AEC2 is essential for AM development during neonatal life, but also for their maintenance during adulthood (76). Interestingly, even if ILC2s and basophils are the main producers of GM-CSF in the lung (52), their production of GM-CSF seems to be redundant for AMs development (76). It can be assumed that differences in the location of these cells and their interactions with AMs may be the key to this process.

Besides the role of AECs in the development and maintenance of AMs, AECs are also responsible for the regulation of the anti-inflammatory state of AMs (77). For example, signal regulatory protein alpha (SIRP-α) expressed on AMs binds surfactant proteins (SP)-A and -D on AECs to inhibit macrophage activation and phagocytosis (27, 78). Furthermore, CD200-CD200R (AEC-AM) interactions negatively regulate Toll-like receptor (TLR) signalling and reduce the production of pro-inflammatory cytokines by AMs (67), which is essential for maintaining homeostasis. Importantly, during infection, this interaction may lead to desensitization to bacterial TLR ligands, reducing chemokine production and NF-κβ activation on AMs, thereby contributing to increased bacterial susceptibility (79, 80). Other mediators secreted by AEC2 are also important for the developmental biology of AMs including TGF-β and IL-10. AECs can induce integrin-dependent TGF-β activation (81), which allows TGF-β to bind to its receptor on AMs, a process that is important for the maintenance of AMs and their hyporesponsive state (63). IL-10 produced by AEC2s (82) is also involved in ensuring this hyporesponsive state (77) by driving anti-inflammatory functions at mucosal sites (83, 84). In particular, IL-10 signaling on AMs downregulates pro-inflammatory genes and leads to expression of chemokines C-C chemokine receptor type (CCR)5 and CCR1, which may be important to promote their migration to the infection site (85). TLR agonists are able to inhibit IL-10 responsiveness, and maybe in this way, promote the retention of AMs at the infection site and unleash their production of TNF-α (85). Furthermore, the production of IL-10 and PGE2 by AECs can promote the secretion of suppressor of cytokine signaling (SOCS)3 by AMs into exosomes, which are then taken up by AECs and inhibit STAT activation, thus contributing to the control of inflammation (86).

## Innate lymphoid cells group 2

Expansion and activation of ILC2s during neonatal life, which starts with the production of IL-33 by AEC2, correlates with the appearance of AMs (87). ILC2s produce IL-13 during the first days of life and polarize AMs towards an M2 phenotype (87). IL-13 is required for the early polarization and maintenance of AMs, but also MO-AMs, in an M2 state in adult mice (87). Likewise, we have recently highlighted the crucial role that pulmonary ILC2s play in conferring identity and functional polarization to MOs that differentiate into AMs during life (88). In particular, we have shown that after Murid herpesvirus 4 (MuHV-4) infection, a mouse γHV, lung ILC2s exhibit a reduced ability to produce type 2 cytokines in response to HDM, thereby contributing to protection against allergic airway inflammation. Furthermore, MuHV-4 infection triggers GM-CSF production by these lung ILC2s, which orders the differentiation of MOs into AMs, without promoting their type 2 functions. In addition, other study has also described how microbial infections may induce a similar inhibition of ILC2 Th2 properties, leading to a reduction of IL-5 and IL-13 upon exposure to airway allergens (89). In both cases, this inhibition seems to be mediated by IFNγ signaling, a well-known suppressor of ILC2s (90, 91).

## Basophils

Basophils localize in the lung parenchyma, in close proximity to the lumen of the alveoli (52). Lung basophils have a specific imprinted signature compared to their circulating counterparts. They are characterized by IL-6, IL-13, chemokine (C-X-C motif) ligand 2 (CXCL2), TNF-α, Oncostatin M (Osm), and CCL4 expression (52). After birth, epithelial cells produce IL-33 and GM-CSF, which trigger an activation cascade that induces the release of IL-6, M-CSF and IL-13 from basophils. This process contributes to the polarization of AMs towards an anti-inflammatory phenotype. Indeed, depletion of basophils after birth leads to a defect in the development and maturation of AMs, affecting their phagocytic and anti-inflammatory profile.

## Nutrients

In addition to the interaction of AMs with the niche cells, the nutrient composition of the niche itself is decisive in shaping the phenotype and function of the AM population (92). The alveolar niche contains low levels of glucose, which may be important to prevent bacterial outgrowth in the airways (93). This low availability of glucose impairs glycolysis at the niche site and regulates AMs responsiveness to type 2 inflammation (93). Consequently, AMs show limited activation by IL-4 stimulation and helminth infections in comparison to IMs (94). However, glucose levels can change during some inflammatory conditions, such as COPD, asthma and cystic fibrosis and likely facilitate the activation of AMs (92). Similarly, iron levels in the lung can also regulate responses to inflammation. Increased levels of iron are associated with increased pro-inflammatory responses in asthma (95) and COPD (96). AM uptake of iron can protect from oxidative stress during COPD (96), but it can also affect the phenotype and function of AMs. AMs treated with red blood cell-derived iron (hemin) upregulate TNF-α and CXCL8, and downregulate IL-10 (97). Furthermore, hemin-treated AMs downregulate the expression of human leucocyte antigen DR (HLA-DR), TLR4, CD14 and Macrophage receptor with collagenous structure (MARCO), which in turn affects the phagocytosis and response to *Haemophilus influenzae* (97). On the other hand, iron deprivation can also change the metabolism and function of AMs, as it disrupts the tricarboxylic acid (TCA) cycle and augments LPS-induced itaconic acid production, which has anti-inflammatory and anti-microbial properties (98). Lipids are other abundant regulatory component of the alveolar niche that play a role in lung disease (99, 100). For example, ceramide and sphingosine can inhibit AMs phagocytosis of apoptotic cells and promote lung injury during emphysema (101).

The alveolar niche provides the necessary signals to control the reactivity of AMs during homeostasis, in processes such as clearance of apoptotic cells, or to prevent any undesirable inflammatory response (102). Interestingly, at steady state, AMs do not express TLR9 and have a higher threshold for activation by CpG than IMs (102). The niche instructs AM hypo-responsiveness regardless of their origin, as MO-AMs also become unresponsive to CpG stimulation (103). In contrast, IFNγ treatment triggers a TLR9-dependent response in AMs, and its persistence may contribute to inflammation caused by dysregulated TLR9 activation (102). The aging microenvironment may also affect greatly AM phenotype and response to infection and inflammation (104). As an example, increased levels of hyaluronan in the BAL fluids of aged mice, which interacts with CD44 on AMs, seems to be linked to the reduced proliferation capacity observed on AMs (103, 104).

## Monocyte contribution to the resident alveolar macrophage population during life

A broad range of literature has shown that AMs are able to self-sustain independently of MOs (57, 59–61, 105). AM turnover has been studied using different cell labelling techniques *in vivo* and parabiosis experiments (9, 61, 63). At steady state, AM turnover is slow (106), and BrdU labelling experiments show that ~20% AMs become BrdU^+^ after 1 week. However, single cell analysis of AMs at steady state has revealed the presence of a specific cluster of AMs with increased enrichment in proliferation and cell cycle pathways (107), which raises the question whether AM maintenance relays on a specific precursor population. The long lifespan of AMs seems to be in part mediated by the upregulation of the pro-survival molecules Akt and ERK upon activation of SHP-2 by binding of surfactant proteins (SP-)A and SP-D to SIRP-α on AMs (106). Besides, accumulating evidence has shown an important contribution of MOs to the AM pool beyond the aging process (56). Indeed, MO contribution can reach more than 70% after approximately 8 months (60), and this contribution can be accelerated during inflammatory events that may trigger the depletion of the niche (45, 108–110)(**Figure 2**). It is speculated that even at steady state, low levels of inflammation may be the driver of the replacement of resident AMs with MO-AMs. However, the mechanisms behind this phenomenon are still unknown (75, 111).

In humans, the first evidence of MO-derived AMs came from studies on bone marrow transplant patients (112). It was then showed that resident AMs from the host were progressively replaced by MO-AMs from the donor (112), which is consistent with the results from other studies using chimeras generated by full irradiation in mice (113). However, the study of the contribution of MO-AMs (or at least host-derived cells) to the resident AM pool in other settings, such as in lung transplant patients, has led to conflicting results. A study in HLA-mismatched donor-recipient transplant patients showed that donor AMs account for more than 87% of the total AM population up to 3.5 years after lung transplantation (114). These results are confirmed by studies in mice using parabiosis experiments between CD45.1^+^ and CD45.2^+^ *Ccr2* ^-/-^ mice, showing that less than 5% AMs originate from circulating MOs at steady state (59, 61). However, a transplant lung may be far from the “steady state”. For example, another study found an early decrease in donor AMs after transplantation that may lead to the recruitment of MOs differentiating into AMs resulting in the generation of a chimeric AM population that persists for the long-term (115). Subsequent studies in lung transplant patients also confirmed the major contribution of host derived AMs to the donor AM pool over time (116, 117). Unfortunately, one of the limitations of all these studies is the small sample size (~15-4 patients respectively) and the high variability observed between patients, which may be due to different ages and health conditions. It is therefore not possible to determine precisely under which conditions donor AMs can be more easily replaced by host derived AMs. However, it is sufficient to highlight the important contribution of MOs to the AM population over time, which may be higher than expected. MO differentiation into AMs creates a continuum of transition states that leads to intermediate populations. Transitioning MOs progressively downregulate MO markers, such as CD11b, and start upregulating AMs markers, such as SiglecF, a process that may take up to 7 days to be detectable in the transitioning population (118). It is therefore important to take this process into account and to carefully examine potential contribution of MOs to the AM pool when analyzing early time points after the onset of inflammation. For example, if the contribution of MOs to AMs after LPS challenge is examined after 3-5 days and any cells expressing CD11b or Ly6C are excluded from the AM gate (119), the MO-AM transition population could be excluded, leading to the conclusion that MOs do not contribute to the final AM pool in this context of inflammation. Furthermore, although in some cases the resident AM population is able to fully recover to initial baseline levels (59, 106, 107), potential overcrowding of the lung with MO-AMs after inflammation should be considered, as these populations may still contribute to the final resident pool (45, 109).

The contribution of MOs to the AM pool is highly dependent on the type of depletion and the turnover rate of AMs and IMs in the lung (5, 113, 120). Along with this, changes in the alveolar environment and its composition may also determine how the AM pool is replenished after depletion. Guilliams and Scott proposed a model in which niche accessibility, availability and precursor plasticity would determine the origin of tissue-resident macrophage populations (121). In the context of the alveolar niche, accessibility of MOs to the niche site could be a limiting factor. However, at steady state, Ly6C^+^ MOs are able to circulate the lung without contributing to resident macrophages (122). Furthermore, the particular architecture of the alveolar niche, in which 2 out of 3 alveoli remain devoid of AMs at steady state (38) may imply that niche availability, if seen as physical space, may not be the solely determining factor for MO engraftment in this particular organ. On the other hand, the combination of chemokines and cytokines released during inflammation, and the increased availability of trophic factors during apoptosis of resident AMs may allow incoming MOs to differentiate more easily into AMs.

Similar to FL-MOs differentiation into embryonic AMs (EM-AMs) during development, the differentiation of BM-MOs into MO-AMs is also dependent on GM-CSF and TGF-β signaling (59, 63)(**Figure 3**). Furthermore, other cytokines, such as IFNγ may also contribute to the replacement of AMs with MO-AMs. Indeed, instillation of IFNγ is able to reduce AM counts only in IFNγ-receptor functional AMs (118). Similarly, MO differentiation into macrophages is IFNγ-dependent during *Toxoplasma gondii* infection in the peritoneum (123), or during experimental autoimmune encephalomyelitis (EAE) in the central nervous system (124). Once BM-MOs differentiate into MO-AMs and gain access to the alveolar niche, these cells will have to outcompete EM-AMs in their repopulation and renewal capacity in order to persist. The distinct proliferation capacity of MO-AMs and EM-AMs may be due, on the one hand, to a different accessibility (72) and/or response (70, 125) to GM-CSF but also, on the other hand, to intrinsic differences in their metabolism (109). For example, although FL-MOs respond better to GM-CSF than BM-MOs (70, 125), differences in the accessibility of BM-MOs to elevated levels of M-CSF, as described in the peritoneum, may allow these cells to increase their proliferation and replace embryonic peritoneal macrophages (72). Furthermore, enhanced glycolytic activity in MO-AMs seems to help these cells to outcompete EM-AMs after Influenza infection (109)

In COVID-19, a defect on GM-CSF-dependent differentiation of MOs into AMs appears to be responsible for the exacerbation of local inflammation and the worsening of health status (126). In this case, local intranasal administration of GM-CSF may accelerate the progression of macrophage populations into a niche-imprinted AM phenotype (126), a concept that was first demonstrated for influenza infection (127). It is important to consider that although GM-CSF plays a key role on lung homeostasis, it is also associated with the induction of a pro-inflammatory program on CCR2^+^ MOs (128), which makes it a double sword mediator of both tissue homeostasis and inflammation. This duality has led to confronting approaches in the treatment of COVID-19, with some approaches proposing to block GM-CSF systemically and others to administer it locally in the lung (129). Besides the obvious importance of the administration route, timing of the administration and lung status at that time may also be critical in the outcome (126, 129).

Different models coexist to explain how distinct AM populations may contribute to inflammation and its resolution. In this context, Watanabe et al. have proposed a passive and active model of tissue repair. In the ‘passive model’, MO-AMs progressively differentiate into homeostatic resident AMs in parallel with tissue regeneration. Alternatively, MO-AMs die during the resolution phase and allow the recovery of tissue-resident AMs (130). Indeed, newly differentiated MO-AMs have increased expression of Fas compared to resident AMs (106). Consequently, Fas activation by the increased levels of FasL, both soluble and membrane-bound (106, 131) may make MO-AMs more susceptible to undergo apoptosis compared to resident AMs. In the ‘active model’ of tissue repair, MO-AMs may undergo specific transcriptional programs induced by integration of signals from the injured tissue (130). For example, in the liver, release of IL-1β during necroptosis of Kupffer cells in *Listeria monocytogenes* infection triggers IL-33 secretion by hepatocytes, and IL-4 secretion by basophils, which instruct MO-derived macrophages to proliferate and shift towards a reparative phenotype (132).

## Alveolar macrophage identity: How much of their origin signature persists at the niche site?

Precursors of macrophages with, YS, FL or BM origin are able to colonize an empty alveolar niche (125). However, mature macrophages from other tissues fail to be fully reprogrammed by the tissue microenvironment (125, 133). Tissue residence time has been shown in this case to be one of the factors limiting the ability of mature macrophages (133), but also precursors of macrophages, such as YS-MACs (62), to differentiate into other macrophage populations.

The niche environment governs the expression of tissue-specific macrophage phenotype and function. However, tissue-resident macrophage populations have unique poised and active enhancers that both reflect developmental origin and tissue specificity (133). AMs of YS, FL and BM origin are almost identical, transcriptionally and functionally, and in all cases are able to prevent alveolar proteinosis (125, 134). However, AMs of different origin preserve a small portion of origin-related marks independently of the niche environment instruction, even at steady state (12).

The relevance of origin to the phenotype and function of AMs has been debated over the last decade (77). Different opposite phenotypes have been ascribed to AMs during lung immune responses to allergens, infections and cancer. This raised the question, whether the so-called plasticity of AMs could be explained in some cases by the heterogeneity of AMs that populate the lung during these events (135, 136).

One of the main EM-AM restricted genes is MARCO (137). MARCO is an scavenger receptor that plays an important role not only in phagocytosis, but also in the regulation of TLR-mediated responses (138, 139). Thus, reduced expression of MARCO in MO-AMs may affect their functionality during infection. Furthermore, EM-AMs have increased expression of DNA replication and cell division genes, which could favor their persistence in the niche (125). However, fully differentiated AMs of different origin have a similar turnover both in mice and in human (70, 125) which would imply that MO-AMs could potentially persist for the long term, once established into the niche. In contrast to EM-AMs, MO-AMs overexpress genes associated with the antiviral response and have a tendency to increase expression of genes associated with antigen presentation (125). In line with this, human MO-AMs also show an IFNy-induced signature (71). Furthermore, MO-AMs show enrichment in pathways involved in immune signaling, inflammation, glycolysis and arginine metabolism (107). However, in this case, as most of the differences are observed at the peak of inflammation, it is difficult to say whether this is due to an early undifferentiated state or whether these changes persist later, when a new challenge arises.

The functional relevance of the transcriptomic differences observed between EM-AMs and MO-AMs seem to become apparent during infection and inflammation. Accumulation of MO-AMs in aging mice promotes increased morbidity and mortality and impaired lung function after influenza infection (109). In addition, MO-AMs contribute to the development of lung fibrosis (137, 140, 141). Indeed, MO-AMs that arise during bleomycin-induced fibrosis are enriched in profibrotic genes compared to EM-AMs (140), and localize in close proximity with fibroblasts, where they exert their pro-fibrotic effects (141). These MO-AMs persist in the long term (at least up to 10 months) after the resolution phase, and although they start to resemble EM-AMs, they retain more than 300 differently expressed genes compared to EM-AMs (140). Nevertheless, it is unknown if these differences at the transcriptional level, or possible imprinted marks at the epigenetic level, may allow these cells to respond in the same way during a recurrent inflammatory event.

Although the origin may indeed dictate different functional profiles on AMs, in some cases, heterogeneity of AMs seems to be independent of their origin and location. As such, fungal infections induce the appearance of two different population of AMs, which can be separated by their expression of CXCL2, a known chemoattractant of neutrophils (25). AMs expressing CXCL2 are *bona fide* sentinels, metabolically active and with high phagocytic capacity. On the other hand, AMs lacking CXCL2 are metabolically more quiescent than homeostatic AMs, and exhibit an anti-inflammatory profile. These CXCL2^-^ AMs produce IL-10 and complement component 1q (C1q), which seems to be important in programming and sustaining anti-inflammatory AM functions (25). Interestingly, the IL10 and C1q production seem to be intrinsic while CXCL2 expression depends entirely on the microenvironment (25). Similarly, resident AMs maintain their M2-like phenotype during inflammation while upregulating proteasome and immune response genes during peak inflammation (107). These examples highlight the functional plasticity of resident AMs populations, which does not fit with the initial M1/M2 dichotomy. In human also, beyond the differences linked to the ontogeny of AMs or to the type of inflammation, part of the heterogeneity of AMs seems to be due to the existence of sub-compartments within the niche with defined programming states (142). Indeed, a recent study has shown several superclasters of AMs with distinct phenotypic properties, conserved between healthy individuals and those suffering from mild cystic fibrosis. These observations may suggest the presence of sub-niches of AMs with different spatial and functional specializations even at steady state (142).

## Alveolar macrophage identity after infection, when can we talk of imprinting?

### Defining priming, trained immunity and tolerance as distinct immunological processes

**Figure 4:**
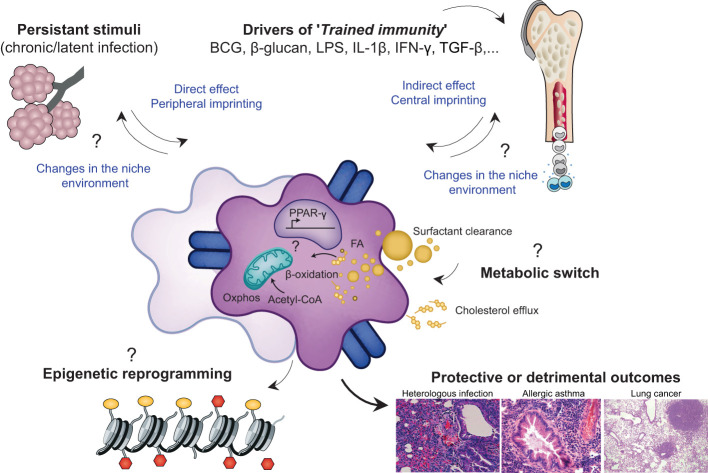
Environmental factors imprint AM phenotype, metabolome and epigenome leading to diverses functional consequences in the context of health and disease. '*Immune imprinting*' refers to an epigenetic, metabolic and functional long-term reprogramming of innate immune cells including EM-AMS or MO-AMS, that results in an altered response to subsequent unrelated triggers. This reprogramming can take place at the level of the inflammed niche (peripheral imprinting) and/or at the level of the progenitor stem cells in the bone marrow (central imprinting) explaining the possible very long term effects of an infectious event, despite the short half-life of immune cells such as MOS. The drivers of this immune imprinting are multiple Most of the currently known environmental factors are the BCG vaccine, microbial products such as LPS, β-glucans, and certain metabolites or pro-inflammatory cytokines. These stimuli can be transient and responsible for processes of trained immunity or long-term tolerance or they can be persistant and thus permanently/recurrently educate AMs. These central and/or peripheral stimuli trigger a cascade of intracellular signaling pathways that can lead to profound alterations in the metabolic and epigenetic profile of the cell The persistence of epigenetic marks and thus the long-term modification of chromatin accessibility at certain loci determines the phenotypic signature of imprinted cells and subsequently, their behaviour in heterologous inflammatory challenges. Interestingly, the imprinting of a given cell could lead to secondary alterations in the cells of the niche and thus modify the cross-talk established between the resident cents of the alveolar niche over the long term. Overall, the history of infection can shape the functional properties of AMs with consequences, depending on the context of imprinting and the context of challenge, being positive or detrimental for the host.

‘Immune imprinting’ refers to an epigenetic, metabolic and functional long-term reprogramming of innate immune cells, which result in an altered response to subsequent unrelated triggers (143). MOs and AMs can be shaped by this immune imprinting and undergo different ‘adaptive’ programs that determine the orientation and the intensity of their response to subsequent stimulation (**Figure 4**). These ‘adaptive’ programs have been defined as cell differentiation, priming, tolerance and trained immunity, and their induction depends on the magnitude of the stimulus, but also on its duration (144). ‘Cell differentiation’ refers to the progression from a precursor or immature cell to its mature counterpart, a concept that corresponds to the differences discussed earlier between EM-AMs and MO-AMs, given their different origin. ‘Priming’ happens when functional changes induced on MOs or AMs after a first stimulation, are still transcriptionally active at the time of secondary stimulation or infection. Thus, the impact of a second challenge in primed cells is often additive or synergistic with the original stimuli (144). In contrast, ‘trained immunity’ and ‘tolerance’ require the immune activation status of MOs or AMs to return to basal levels before secondary stimulation, while the epigenetic alterations persist. Although trained immunity refers to gain of function or enhanced immune response, tolerance refers to loss of function or decreased immune response. Whereas these definitions may help to create consensus, they focus only on the induced changes and the activation status of a given cell, without considering the impact that persistent changes in the niche environment and/or other cells may have on the activation of these cells upon secondary stimulation. For example, low doses of LPS induce trained immunity on AMs in a type I IFN-dependent manner that requires AMs re-wiring metabolism towards fatty acid oxidation and glutaminolysis (145). These trained AMs produce increased levels of IL-6 upon *ex-vivo* secondary stimulation with bacteria (145). However, the transfer of trained AMs into naive mice, followed by subsequent bacterial challenge, not only fails to protect the mice, but it increases bacterial loads and inflammation. Protection is only achieved when LPS treatment is carried out before infection in the same mouse (145). This indicates that the niche environment and/or other cells affected by LPS exposure also contribute to the response of trained-AMs to bacteria.

We have recently shown that the recruitment into the lung of MOs with a regulatory profile during γHV infection plays an important role in the control of viral-induced immunopathology (146). On the contrary, the recruitment of MOs during Influenza infection and their contribution to the AM pool is associated with a proinflammatory phenotype that exacerbates disease severity and immunopathology (109, 147). These differences highlight that not only the origin but also the functional education of MOs and MO-AMs are relevant in the context of infections and have an impact on disease outcomes. Furthermore, it is important to consider that immune imprinted changes by the same infection can trigger either detrimental or protective responses depending on the context and the type of exposure to heterologous challenges. For example, imprinted MO-AMs present after Influenza infection confer bacterial protection on an IL-6-dependent manner (110), whereas they increase susceptibility to a secondary Influenza infection in an IL-6-independent manner (109).

Trained immunity has mostly been described for NK cells, DCs, MOs and macrophages, and alterations in the metabolism of these cells may be seen as adaptive mechanisms to the changing demands of the environment (148, 149). AMs, as innate cells that continuously integrate environmental signals, are particularly exposed to various training effects. However, evidence for the persistence of epigenetic marks underlying changes in the transcriptomic and functional program of AMs is still being investigated. Indeed, although many studies report long-term phenotypic alterations of AMs in different contexts of inflammation, it is not always easy to distinguish between ‘trained immunity’ and ‘priming’, particularly in cases such as chronic and/or latent infections. However, it is interesting to highlight and understand how the history of infection, exposure to particular microbial products or specific insult, can alter the functional properties of AMs in the long term and potentially affect heterologous immune responses in the lungs.

## Peripheral or central imprinting: How does immune reprogramming affect the balance between health and disease?

The existence of long-lasting effects induced in myeloid cells by microbial products such LPS or beta-glucans or vaccine (*Bacillus Calmette–Guérin* (BCG)) has long been an enigma, as mature myeloid cells such as MOs and DCs have a relatively short half-life. How the trained immunity is maintained in myeloid cells for months or even years has long remained a mystery. Recent research has provided some insights. Thus, innate immune memory in myeloid and haematopoietic stem cells (HSCs) has been demonstrated in response to BCG (150), β-glucan (151) or Western diet (152) suggesting the existence of a ‘central trained immunity’ through HSC reprogramming. The consequences of a central imprinting in long-lived bone marrow progenitors imply that mature cells derived from these progenitors may acquire specific functional profiles when recruited to inflamed organs. Furthermore, central imprinting could have major consequences in shaping local niches, in which recruited MOs may replace resident macrophage populations and persist in the long term, as it is the case for AMs.

One of the first reported mechanisms of trained immunity on MOs was described for *Candida albicans* (153). This infection induces training on MOs that is sufficient to protect Rag1-deficient mice from reinfection (153). The effect is mediated by the recognition of β-glucans, which is dependent on dectin-1 and the complement receptor 3 (153). Trained MOs show profound changes in their transcriptomic profile, which correlates with epigenetic changes in the methylation of the DNA packaging protein Histone H3 (H3K4me3). These trained MOs express increased levels of M1 markers, including TNF-α, IL-6, TLR2 and TLR4, but also M2 markers such as MRC1 and CD163, and a concomitant decrease in Arg1 expression (153). Together with β-glucans, BCG has been one of the most studied inducers of central trained immunity. Indeed, BCG is known to trigger the expansion of HSCs, to promote myelopoiesis, but also to induce training in MOs and macrophages by imprinting a new transcriptomic and epigenetic profile (150, 151). The newly acquired functional profile protects against tuberculosis (150), but also against other pathogens such as Influenza, Yellow fever vaccine virus, *Candida albicans*, *Schistosoma mansoni*, as well as against chemotherapy-induced myelosuppression (151).

Further scientific evidence of central imprinting and mechanistic keys were subsequently provided by de Laval and colleagues who demonstrated that experimental injection of LPS induces cryptic open chromatin regions in HSCs in a C/EBPb-dependant manner which directs an improved response to secondary infection (154). The scientific support for central imprinting following viral infections is poorer at present. Indeed, although widely suggested in COVID 19, evidence of epigenetic alterations in myeloid progenitors and HSCs is not yet published. While SARS-coV2 infection disrupts the haematopoietic balance and induces a bias towards neutrophil differentiation (155), it would be interesting to define whether this phenomenon is based on lasting epigenetic alterations in HCS and/or myeloid progenitors. Therefore, the persistence of potential epigenetic marks at this level could be related to the commonly known ‘*long-COVID’* condition.

The induction of trained immunity is driven in most cases by pathogen recognition receptors (PRRs) and the secretion of inflammatory mediators during infection, such as alarmins, cytokines or interferons (156, 157). Indeed, stimulation of mature macrophages with TLR agonists, LPS, IL-4, IFNγ, TNF, TGF-β and IL1-β leads to persistent epigenetic changes (158). However, the type of imprinting can be highly dependent on the dose, the severity of the infection or inflammation, and the combination of particular mediators. For example, while low doses of LPS or *Candida albicans* can induce trained immunity (145), high doses may induce tolerance (159). In the same line, IFNγ plays a critical role in the induction of trained immunity (149), as it is required, for example, in the reprogramming of HSCs in the bone marrow after BCG infection (150). However, its effect seems to be widely dependent on its concentration and the presence of other mediators. During pneumococcal pneumonia, IFNy signaling is also responsible for the upregulation of MHCII and CXCL9 on MO-AMs and resident AMs (118). However, only MO-AMs have increased expression of PRRs (TLR2, TLR4) and phagocytic receptors (CD93) (118). Furthermore, IFNγ is able to partially restore the metabolic defects on immunotolerant MOs in sepsis by promoting glycolysis (159). On the other hand, IFNγ secretion during severe Influenza infection has been associated with immune-paralysis state in AMs (160). IFNγ treatment, while enhancing antigen presentation, reduces AM phagocytosis, that correlates both *in vitro* and *in vivo* with the downregulation of MARCO (12, 138). Similarly, severe pneumonia also causes an immune-paralysis state in resident AMs that persists even after 1 month (119), mimicking the states of immunosuppression observed in human patients with hospital-acquired pneumonia. This state can develop after different bacterial (*Staphylococcus aureus* and *Escherichia coli*) or viral infections (119). In this case, the AM phenotype is the consequence of tolerogenic training by immunosuppressive signals that is dependent on SIRP-α signaling in AMs during the resolution phase of the primary infection, resulting in reduced clearance of bacteria in subsequent infections (119).

Different cells can participate in the imprinting of MOs and AMs through IFNγ secretion in both the bone marrow and the alveolar niche, including NK cells (160) and CD8 T cells (161), respectively. Adenovirus infection has been the first infection described to induce local imprinting of resident AMs, mediated by the adaptive arm of the immune system. These trained AMs are characterized by increased glycolytic metabolism, high MHCII expression and enhanced neutrophil chemokine responses, contributing to better immunity against bacterial infections (161). In this context, the bidirectional crosstalk between trained immunity and adaptive immunity is still under investigation. However, AM imprinting leading to changes in antigen presentation and cytokine secretion is likely to have important consequences for AM crosstalk with tissue-resident memory T cells and their activation, which could affect deeply immune responses in the lung.

It is interesting to note that local imprinting may be specifically restricted to the niche area affected by the initial infection. For example, *Streptococcus pneumoniae* is able to induce long-lasting specific metabolic and transcriptional changes in AMs that reside in the lung lobule initially infected, where they confer protection to other bacterial infections (162). Many questions remain about the maintenance and persistence of immune imprinting. For example, it is unclear if all or only some epigenetic marks are transmitted to the cells progeny, and it is unknown how the niche instruction and metabolism can act not only in inducing epigenetic changes in niche cells but also on reverting them.

The training effects of different microorganisms play important roles not only on educating the immune system to improve responses to incoming pathogens but also to regulate their reactiveness and promote tolerance. Commensal bacteria seem to play a key role in the education of mucosal immunity and in particular in the responsiveness of macrophages against viral infections (163, 164). Indeed, antibiotic-treated mice have increased susceptibility to influenza that correlates with the presence of lung macrophages deficient in type I and II IFNs, and in antiviral defense genes, resulting in a reduced ability to limit viral replication (163). Furthermore, *in vivo* challenge of ‘*specific pathogen free*’ (SPF) mice with *Staphylococcus aureus*, a common pathogen in the respiratory tract, is able to polarize incoming MOs into M2 MO-AMs that inhibit influenza-mediated tissue damage and inflammation in a TLR2 and IL-13-dependent way (165).

While the imprinting of the innate immune system, and of AMs in particular, is of major importance in the subsequent responses developed against infectious agents, his imprinting can also profoundly shape non-infectious and unrelated immune responses. These include respiratory allergic responses as well as anti-cancer lung immunity. Indeed, long before the emergence of the concept of ‘trained immunity’, the ‘hygiene hypothesis’ had already established an epidemiological link between childhood exposure to microbial products, such as LPS, and subsequent protection against the development of atopy or respiratory allergies (166). However, the detailed understanding of the mechanisms is not complete and the involvement of trained immunity (central and/or peripheral) in this process is not elucidated and is probably context-dependent. Interestingly, we have established in mice the importance of exposing a naive immune system to symbiotic viruses to prevent the development of allergic airway inflammation. More precisely, we have shown that a gammaherpesvirus infection provides long-lasting protection against HDM-induced allergic airway inflammation, by the replacement of resident AMs with MOs with an imprinted regulatory phenotype (45). These regulatory MO-AMs are characterized by increased MHC-II, Sca1 and IL-10 expression (45) and display a phenotype similar to that of MOs observed after *Toxoplasma gondii* infection in the intestine (160). This protection is characterized by the inhibition of lung eosinophilia and the reduction of Th2 cytokine production, both in the lung and in the dLN, which correlates with impaired DC activation. Importantly, virus-imprinted AMs play an essential role in this phenotype, as the adoptive transfer of MO-AMs isolated from MuHV-4 infected mice into naive recipient mice is sufficient to confer protection against HDM-induced allergic airway inflammation. Whereas gammaherpevirus infections are associated with protection against allergic responses (45, 167, 168), most viral infections are correlated with an amplification of the pulmonary Th2 response. Indeed, multiple epidemiological studies have linked neonatal viral infections such as human metapneumovirus (HMPV), and rhinovirus (RV), and in particular, respiratory syncytial virus (RSV) infections, to the subsequent development of atopy and allergic asthma in adulthood [recently reviewed in (169)]. Several mechanisms have been showed in the mouse model to contribute to this increased susceptibility to allergic disease later in life. For example, RSV infection promotes a Th2-like inflammatory response in the lung of newborn mice that induces a Th2-like effector phenotype in regulatory T cells and attenuates tolerance to an unrelated antigen such as allergen (170). Furthermore, RSV-specific CD8+ T cells induced in neonatal mice showed a defect in IFN-γ production (171). This lack of IFN-γ production leads to a defective M1 macrophage response (172). Conversely, the increased production of IL-33 and the subsequent overactivation of ILC2s (173) reinforce the dominance of type 2 immunity. This general skewing towards type 2 inflammation could have a direct effect on asthma susceptibility but could also have an indirect effect on the Th1/Th2 balance later in life by promoting a M2 polarization on AMs and MO-AMs for the long term. In particular, we have recently published a key role for ILC2s in conferring AM identity and polarisation after a virus-induced depletion of the alveolar niche (88). Deciphering the potential contribution of ‘M2’ AMs (or MO-AMs), trained early in life by activated ILC2s, in long-term sequelae like asthma, could be of major importance in future research.

Another example of the role of imprinted-AMs in ‘non-infectious immunity’ is their importance in the development of cancer. Indeed, given the ambivalent role of macrophages in cancer, it is questionable what influence different imprinted programs on AMs may have in the context of anti-tumor immunity. For example, at steady state, lung resident macrophages including AMs provide a pro-tumorogenic niche for tumor cells development (174). In contrast, epigenetic rewiring of HSCs following microbial or nanobiologics exposure enhances myelopoiesis and macrophage activation, which improves therapeutic outcomes in a mouse melanoma model (175). In addition, a recent study has shown the positive effects of experimental trained immunity in lung macrophages to control metastasis establishment (176). In particular, intraperitoneal administration of whole β-glucan particles to mice, followed by intravenous injection 7 days later of lung Lewis carcinoma cells, induces BM myelopoiesis and MO activation, leading to the control of metastasis. In this case, the alveolar niche is not depleted, but the trained-MOs differentiate into MO-IMs without contributing to the AM pool, potentially explaining why only IM-MO are associated with reduced tumour development (176). The specific contribution of virus-trained AMs in anti-tumor immunity has been recently investigated in an elegant study published by Wang et al. (177). Using mouse models of influenza and lung metastatic tumors, they showed that influenza enhanced the phagocytic and cytotoxic functions of AMs in an IFNγ-dependent manner, leading to lasting resistance to tumor-induced immune suppression (177). Although lung tissue-resident trained AMs, induced by influenza infection, have been shown to exert long-lasting tissue-specific antitumor trained immunity, their beneficial *versus* detrimental role could be context-dependent, given that enhanced responses must be tightly regulated to avoid excessive carcinogenic inflammation (178).Therefore, further research in the future could shed light on the unappreciated role of tissue-specific antitumor trained immunity mediated by embryonic self-sustaining AMs *versus* MO-derived AMs.

Many questions remain open at present when it comes to demonstrating the particular causal link between the AM training effect and individual differences in susceptibility *versus* resistance to disease development. As such, knowledge gaps persist in understanding the specific contribution of trained EM-AMs *versus* MO-AMs to the development of context-dependent lung disease. How and where these AMs are imprinted? What is the relative importance of central imprinting compared to local education? What are the contributors to this imprinting and to what extent this imprinting is associated to reversible metabolic and epigenetic regulation that could be targeted for therapeutics intervention? Moreover, there is still limited information on the impact of trained immunity on the adaptive immune response, both in settings of acute and chronic inflammation. Future research should clarify whether specific drivers of trained immunity may differentially affect subpopulations of T cells and disrupt the delicate Th1/Th2 balance and how it may impact the development of certain inflammatory conditions.

## Conclusion

In conclusion, the accumulated information on AMs shows the complex roles that these cells play, not only in maintaining lung homeostasis and ensuring efficient respiratory exchange but also in protecting this large mucosal surface from pathogen invasion. Multiple intrinsic and extrinsic factors shape the functional properties of AMs and therefore influence the responses developed by these cells to different types of stress. These factors include the ontogeny of AMs, which determines part of the plasticity and reactivity of these cells, and the microenvironment of the alveolar niche. This local imprinting is mediated by the close interactions with niche immune (basophils, ILC2s, T cells) and non-immune (epithelial cells) cells, but also by the composition of the alveolar niche in nutrients, ions, and (commensal) microorganisms. In addition, a central imprinting, occurring at the level of bone marrow progenitors, may also influence the phenotype of MOs infiltrating the airways that differentiate into AMs. These changes, whether or not associated with long-term metabolic and epigenetic reprogramming, contribute to the functional profile of AMs and to the immune responses developed in the lung. While many questions remain, understanding the context-dependent conditions that determine the reversible polarization of these cells is of fundamental importance to better understand the mechanisms underlying the development of respiratory immunopathology, but above all, to define targeted therapeutic strategies to restore the immune balance.

## Author contributions

LR-R wrote the manuscript. LG edited the manuscript; BM edited the manuscript and prepared the figures. All authors contributed to the article and approved the submitted version.
